# Association of assisted reproductive technology with adverse pregnancy outcomes

**Published:** 2015-03

**Authors:** Zhang Jie, Ding Yiling, Yu Ling

**Affiliations:** *Department of Obstetrics and Gynecology, The Second Xiangya Hospital, Central South University, Changsha, Hunan Province, People’s Republic of China.*

**Keywords:** *Infertility*, *Assisted reproductive technique*, *Pregnancy complications*

## Abstract

**Background:**

More and more infertile patients have accepted the assisted reproductive technique (ART) therapy. Concerns have been raised over an increased risk of adverse maternal outcomes in ART populations as compared with natural conception (NC).

**Objective:**

The aim was to improve the ART in clinicial work and to reduce the incidence of pregnancy complications in ART group according to analyzing the reasons of high incidence of pregnancy complications in ART group, comparing the incidence of pregnancy complications in different controlled ovarian hyperstimulation (COH) programs and evaluating the effects of ART which attribute to adverse pregnancy outcomes.

**Materials and Methods:**

In this prospective population-based cohort study,3216 pregnant women with gestational age ≤12 weeks, regular antenatal examination,and ultrasound identification of intrauterine pregnancy were enrolled from January 2010 to June 2013. According to having ART history, the participantswere divided into two groups: ART group (contains fresh embryo transfer group or frozen-thawed embryo transfer group) and NC group. We compared the incidence of pregnancy complications between different groups and evaluated the factors which could affect the occurrence of these complications.

**Results:**

When compared to NC group, significantly increased rates of gestational diabetes mellitus (GDM) (p<0.01), preeclampsia (PE) (p<0.01) and intrahepatic cholestasis of pregnancy (ICP) (p˂0.01) were observed in ART group. There was no significant difference in the incidence of birth defect between the two groups (p=0.07). Multiple pregnancies and Gonadotropin (Gn) were risk factors in GDM, PE, and ICP. The exogenous progesterone treatment had no effect on GDM, PE or ICP.

**Conclusion:**

ART increases the risk of adverse maternal complications such as GDM, PE and ICP. The dosages of Gn should be reduced to an extent and the number of embryo implantation should be controlled. Exogenous progesterone treatment is safe.

## Introduction

With the pace of life accelerating, work pressure increasing, environmental pollution worsening and the concept changing, the incidence of infertility in China is approximate 10%. It rises significantly compared with 2-5% in 1980s (1, 2). With the controlled ovarian hyperstimulation (COH) technique improving and assisted reproductive technique (ART) being sophisticated and extensive, more and more infertility patients have accepted the ART therapy and met their pregnancy requirements (3, 4). Concerns have been raised over an increased risk of adverse maternal outcomes like gestational diabetes mellitus (GDM), preeclampsia (PE) and intrahepatic cholestasis of pregnancy (ICP) in ART populations as compared with natural conception (NC) group.

As we all know, most infertility patients need exogenous administration of gonadotropin releasing hormone agonist (GnRH-a), gonadotropin (Gn) and other drugs in order to get the COH treatment before fresh embryo transfer, which is one kind of ART procedures. Some infertility patients get frozen-thawed embryo transfer after hormone replacement cycles and need exogenous estrogen after transfer to promote the endometrial growth and increase endometrial receptivity. All of the patients who get fresh or frozen embryo transfer will get exogenous progesterone treatment at least for 28 days to prevent miscarriage. The treatment patterns include oral intake, intramuscular injection, vaginal suppository and so on. The exogenous progesterone treatment will enhance in case of threatened abortion. 

The exogenous sex hormones, GnRH-a and Gn may affect the sex hormones secretion through hypothalamic-pituitary-gonadal axis and other mechanisms. So the hormone-related complications like GDM, PE and ICP may occur. Moreover, the number of embryos transferred is at least two to ensure live birth rate. There are more twin pregnancies and even triplets pregnancies which lead to the increased incidence of pregnancy complications. Many studies have reported that the incidence of pregnancy complications including GDM, PE, ICP, placenta previa and preterm labor in infertility patients who get ART treatment is higher than pregnant women with natural conception. The findings were consistent with our clinical observations (5-7). But most reports haven’t done the systematic research and could not clarify the cause of the diseases definitely and could not reveal the correlation between COH, exogenous progesterone treatment and pregnancy complications scientifically. 

The objective of this study was to improve the ART in clinicial work and to reduce the incidence of pregnancy complications in ART group according to analyzing the reasons of high incidence of pregnancy complications in ART group, comparing the incidence of pregnancy complications in different controlled ovarian hyperstimulation (COH) programs and evaluating the effects of ART which attribute to adverse pregnancy outcomes.

## Materials and methods

**Subjects**

A prospective population-based cohort study was carried out over three and a half years (January 2010 to June 2013). The study was based on the logbook of the Xiangya Hospital, Central South University, which registers 3216 pregnant women visiting antenatal clinics at the Obstetrics Department. All the pregnant women accepted the regular antenatal examination. Inclusion criteria included: a)first visit time was not later than 12 weeks of gestation; b)identification of intrauterine pregnancy; c) regular antenatal examination. 

Exclusion criteria included: a) chronic hypertension, diabetes, kidney disease, and hepatitis before pregnancy; b) family history of hereditary or chromosomal abnormalities in each of couples. The selected candidates were divided into two groups called ART group (n=428) and NC group (n=2788) according to whether or not accepting ART treatment. ART group contains fresh embryo transfer group and frozen-thawed embryo transfer group. The fresh embryo transfer group included long protocol subgroup, short protocol subgroup, ultra-long protocol subgroup, and the other protocol subgroup. Candidates in ART group accepted embryo transfers. All the candidates registered and filled out the observation sheets.

**Observation contents**

From the first antenatal examination, we recorded the pregnant women’s age, nation, height, body weight and (BMI). For ART group, we needed to record the reasons for infertility, COH protocol, ovulation drugs dosage, spouse’s sperm quality, the pattern and the dosage of exogenous progesterone treatment after embryo transfer and the amount of estrogen use additionally. All the selected candidates should accept the regular antenatal examination including measurement of blood pressure and body weight, detection of liver function and urine protein. We also recorded the pregnant women’s condition during the whole gestation and took appropriate medical intervention and therapy to them for different complications till the termination of pregnancy. At the end, we collected the data of gestational age, delivery way, and complication.

**Diagnostic criteria for related diseases** **GDM**

The Oral Glucose Tolerance Test (OGTT) was taken during 24-28 weeks. According to the recommendations from the WHO Expert Committee, GDM was defined according to fasting venous blood glucose concentration ≥7.0 mmol/L and/or 2 hours post-oral glucose tolerance test venous blood glucose concentration ≥11.1 mmol/L (8).

**Preeclampsia**

Preeclampsia was defined as gestational hypertension and proteinuria and return of all abnormalities to normal by 12 weeks postpartum. Gestational hypertension was defined according to WHO criteria as systolic blood pressure≥140 mmHg and/or diastolic blood pressure ≥90 mmHg and/or higher for the first time after 20 weeks’ gestation (9).

**ICP**

The diagnosis of ICP was based on (I) pruritus of cholestasis, (II) elevated fasting serum bile acids >10 μmol/L (and elevated serum transaminases), (III) spontaneous relief of signs and symptoms within two to three weeks after delivery and (IV) absence of other diseases that caused pruritus and jaundice (10). 

**How to define the number of fetuses**

Singleton pregnancy: there was only one survive embryo which can be assessed by ultrasound technology in the early stage of pregnancy. Twin pregnancy contained the following situations: two embryos were transferred to the woman and two embryos were survived; only one embryo was transferred to the woman but it splits into monozygotic twin pregnancy; multiple pregnancies became twin pregnancy after elective embryo reduction or spontaneous reduction in the second stage of pregnancy. Triplet pregnancy was defined that three embryos were transferred and all three embryos were survived or two embryos were transferred but one of them split into two embryos.

**Confidentiality and the ethics of medical ethics**

This study was reviewed and approved by the Ethics Committee of the Second Xiangya Hospital. Patients had a right to privacy that should not be infringed without informed consent. Identifying information did not be published in written descriptions, photographs, and pedigrees unless the information was essential for scientific purposes and the patient (or parent or guardian) gave written informed consent for publication. Informed consent for this purpose were obtained from all subjects. Identifying details should be omitted if they were not essential, but patient data should never be altered or falsified in an attempt to attain anonymity.

**Statistical analysis**

The results were analyzed with SPSS ver. 19.0 (SPSS Inc., Chi-cago, IL, USA). Numerical variables were expressed as mean±SD (x±s). Categorical variables were expressed as a percentage. Student’s *t*-test was used to ascertain the significance of differences between mean values of two continuous variables. Chi-squares analysis ( ^2^ tests) was performed to test for differences in proportions of categorical variables between two or more groups. Logistic regression analysis was used to evaluate the factors which could affect the occurrence of the pregnancy complications. P-value<0.05 was considered significant.

## Results

The socio demographic characteristics, clinical data, and pregnancy outcomes of the studied pregnant women were summed up in table I. There were 4087 pregnant women who satisfied the inclusion criteria, concluding3306 pregnant women in NC group and 781 pregnant women in ART group. According exclusion criteria, 3216 pregnant women finally enrolled in this study. There were 428 (13.31%) pregnant women in ART group and 2788 (86.69%) in NC group. The follow-up time range was from 1-9 months. 

As shown in table I, the mean age was older in ART group than in NC group (p=0.04). The mean BMI before pregnancy was higher in ART group than in NC group (p=0.01). The Han nationality was the majority ethnic composition in both groups. Women in ART group delivered earlier than women in NC group (p=0.02). The live birth rate was higher in NC group than in ART group (p<0.01). The incidence of GDM, PE, ICP, premature rupture of membranes (PROM) and placenta previain ART group was higher than in NC group (p<0.01). But there was no significant difference in the incidence of birth defect between the two groups (p=0.07).

As shown in Table II, the reasons for getting ART treatment were analyzed. Some women had two or more reasons. 

As shown in Table III, the incidence of different pregnancy complications between different COH programs was compared. The incidence of GDM, PE, and ICP was significantly higher in fresh embryo transfer group than in NC group. 

As shown in Table IV, there was no difference in the incidences of GDM, PE, and ICP between every subgroup and long protocol subgroup comparing separately. As shown in Table V, the differences in the incidence of GDM, PE and ICP with different number of fetuses remained significant. It also showed that multiple pregnancy was a risk factor in GDM, PE and ICP. There was a significant correlation between the number of fetuses and the incidence of GDM, PE and ICP whether adjusted with maternal age, ethnicity and body mass index. 

As shown in Table VI, the differences in the incidence of GDM, PE and ICP with different dosages of Gn remained significant. It also showed that the use of Gn was a risk factor in GDM, PE and ICP. There was a significant correlation between the dosages of Gn and the incidence of GDM, PE and ICP whether adjusted with maternal age, ethnicity and body mass index. As shown in Table VII, there was no difference in the incidences of GDM between three time groups. But the incidence of PE and ICP was significantly higher in the group of treatment time between 28 and 45 days, compared with the other groups. There was not a significant correlation between the exogenous progesterone treatment and the incidence of GDM, PE or ICP whether adjusted with maternal age, ethnicity and body mass index.

**Table I:** Socio demographic characteristics and clinical data

	**ART group n (%)**	**NC group n (%)**	**Comparison ** #	**p-value**
Total (n)	428	2788	-	
Maternal age (mean±SD, year)	32.53±4.02	29.87±3.95	MD 2.63 (1.44-3.82)	0.04
BMI (mean±SD)	21.35±2.74	20.41±1.88	MD 0.94 (0.19-1.70)	0.01
Han nationality (n, %)	421 (98.36)	2746 (98.49)	-	0.83
Gestational age (mean±SD, weeks)	32.35±8.06	36.17±6.68	MD 4.02 (2.04 to 5.99)	0.02
Live birth (n, %)	327 (77.64)	2579 (92.50)	-	<0.01
GDM (n, %)	48 (11.21)	190 (6.81)	OR 1.73 (1.24-2.41)	<0.01
PE (n, %)	45 (10.51)	153 (5.49)	OR 2.02 (1.43-2.87)	<0.01
ICP (n, %)	40 (9.35)	101 (3.62)	OR 2.74 (1.87-4.02)	<0.01
PROM (n, %)	66 (15.42)	262 (9.40)	OR 1.76 (1.31-2.35)	<0.01
Placenta previa (n, %)	35 (8.18)	137 (4.91)	OR 1.72 (1.17-2.54)	<0.01
Birth defect (n, %)	18 (4.21)	159 (5.70)	OR 0.62 (0.37-1.01)	0.07

Independent Student’s *t*-test

MD: mean difference and standard deviation.

# OR: Odds ratio and 95% confidence intervals

**Table II:** Reason analysis of ART group

**Reason for infertility**	**n**	**%**
Tubal factor	320	74.77
Abnormal semen quality	95	22.20
Endometrial factors	14	3.27
Polycystic ovary syndrome	55	12.85
Abnormality sperm-egg binding	6	1.40
Uterine malformations	4	0.93
Absence of maternal blocking antibody	8	1.87
No apparent reason	18	4.21
Age	10	2.34

**Table III:** Complications of different COH programs

	**GDM n (%)**	**p-value**	**PE n (%)**	**p-value**	**ICP n (%)**	**p-value**	**Total**
Fresh embryo transfer group #	41 (12.13)	<0.01	36 (10.65)	<0.01	32 (9.47)	<0.01	338
Frozen-thawed embryo transfer group #	7 (7.87)	0.67*	9 (10.11)	0.09*	8 (8.99)	0.02	89
NC group	190 (6.81)	**---**	153 (5.49)	**---**	101 (3.62)	**---**	2788

Chi-squares analysis (χ^2^ test)

# Fresh embryo transfer group and frozen-thawed embryo transfer group is compared with NC group separately.

**Table IV:** Comparisons between different COH protocols of fresh embryo transfer group

	**GDM n (%)**	**p-value**	**PE n (%)**	**p-value**	**ICP n (%)**	**p-value**	**Total**
Long protocol subgroup	31 (13.14)	---	26 (11.02)	---	20 (8.47)	---	236
Short protocol subgroup#	3 (5.45)	0.16	4 (7.27)	0.62	5 (9.10)	0.80	55
Ultra-long protocol subgroup#	6 (15.00)	0.80	6 (15.00)	0.43	6 (15.00)	0.24	40
Other protocol subgroup#	1 (12.50)	1.00	0 (0.00)	1.00	1 (12.50)	0.52	8

Chi-squares analysis (χ^2^ test)

# Every subgroup is compared with long protocol subgroup separately.

**Table V:** Complications with different number of fetuses in ART group

	**n (%)**	**p-value** ^1^	**Unadjusted OR**	**p-value** ^2^	**Adjusted OR**	**p-value** ^2^	**Total**
GDM							
Singleton pregnancy	18 (7.96)	0.01	2.015 (1.220, 3.328)	<0.01	2.208 (1.304, 3.739)	<0.01	226
Twin pregnancy	25 (13.51)						185
Triplet pregnancy	5 (29.41)						17
PE							
Singleton pregnancy	16 (7.08)	<0.01	2.173 (1.297, 3.640)	<0.01	2.433 (1.407, 4.206)	<0.01	226
Twin pregnancy	24 (12.97)						185
Triplet pregnancy	5 (29.41)						17
ICP							
Singleton pregnancy	13 (5.75 )	<0.01	2.277 (1.324,3.917)	<0.01	2.721 (1.514, 4.891)	<0.01	226
Twin pregnancy	23 (12.43)						185
Triplet pregnancy	4 (23.53)	17

p-value^1^: Chi-squares analysis (χ^2^ test)

p-value^2^: Logistic regression analysis.

OR: Odds ratios and 95% confidence intervals.

Adjusted OR are calculated with correction for potential confounding factors (maternal age, ethnicity, body mass index). Singleton pregnancy group is as a reference,

**Table VI:** The dosages of Gn and pregnancy complications

**Dosages of Gn**			**n (%)**	**p-value** ^1^	**Unadjusted OR**	**p-value** ^2^	**Adjusted OR**	**p-value** ^2^	**Total**
GDM									
		Gn<1500IU	5 (5.68)	0.04	1.625 (1.152, 2.293)	<0.01	1.765 (1.242, 2.509)	<0.01	88
		1500≤Gn<2000IU	12 (10.62)						113
		2000≤Gn<2500IU	16 (16.00)						100
		Gn≥2500IU	8 (21.05)						38
PE									
	Gn<1500IU		5 (5.68)	<0.01	1.469 (1.026, 2.104)	0.04	1.570 (1.094, 2.254)	0.01	88
	1500≤Gn<2000IU		11 (9.73)						113
	2000≤Gn<2500IU		14 (14.00)						100
	Gn≥2500IU		6 (15.79)						38
ICP									
	Gn<1500IU		4 (4.55)	0.04	1.536 (1.050,2.246)	0.03	1.624 (1.117, 2.360)	0.01	88
	1500≤Gn<2000IU		10 (8.85)						113
	2000≤Gn<2500IU		12 (12.00)						100
	Gn≥2500IU		6 (15.79)	38

p-value^1^: Chi-squares analysis (χ^2^ test)

p-value^2^: Logistic regression analysis.

OR: Odds ratios and 95% confidence intervals. Adjusted OR are calculated with correction for potential confounding factors (maternal age, ethnicity, body mass index).

Gn<1500IU group is as a reference.

**Table VII:** The exogenous progesterone treatment time and pregnancy complications in ART group

**Treatment time**		**n (%)**	**p-value** ^1^	**Unadjusted OR**	**p-value** ^2^	**Adjusted OR**	**p-value** ^2^	**Total**
GDM								
	≤28 days	26 (9.63)	0.36*	0.816 (0.561, 1.186)	0.29*	0.820 (0.570, 1.277)	0.30*	270
	28<x≤45days	13 (14.94)						87
	>45 days	9 (12.68)						71
PE								
	≤28 days	23 (8.52)	0.02	0.852 (0.578,1.256)	0.42*	0.858 (0.581,1.280)	0.42*	270
	28<x≤45days	16 (18.39)						87
	>45 days	6 (8.45)						71
ICP								
	≤28 days	15 (5.56)	<0.01	0.836 (0.563,1.236)	0.38*	0.841 (0.568,1.237)	0.39*	270
	28<x≤45days	17 (19.54)						87
	>45 days	8 (11.27)	71

p-value^1^: Chi-squares analysis (χ^2^ test)

p-value^2^: Logistic regression analysis.

OR: Odds ratios and 95% confidence intervals. Adjusted OR are calculated with correction for potential confounding factors (maternal age, ethnicity, body mass index). <28 days group is as a reference.

* mean p>0.05

## Discussion

ART is an important life science, rising in the mid-20^th^ century. It provides adequate technical support for solving the increasing serious problem of infertility. ART involves handling of gametes (eggs, sperms, or both) outside the human body with the ultimate aim of achieving a healthy conception. Commonly performed ART procedures include in vitro fertilization (IVF) with or without intracytoplasmic sperm injection (ICSI), fresh or frozen embryo transfer, IVF with donor oocytes, and intra uterine insemination. 

A number of factors may be responsible for this upward trend of use of ART. With the pace of life accelerating, work pressure increasing, environmental pollution worsening and the concept changing, the incidence of infertile increases year by year. The previous studies found that the most common cause of infertility was the tubal factor. Most of the infertility women have intrauterine operating history (11, 12). Abnormal semen quality is the second main reason for infertility which can affect embryo quality and embryo development. The reasons for infertility still include polycystic ovary syndrome, endometrial factors, age, absence of maternal blocking antibody, abnormality sperm-egg binding, and uterine malformations. Female fertility declines after the age of 35 years, and many women who delay having children until later in life for personal reasons require assisted conception. Nevertheless, there are some infertile women without apparent reason. Many recent studies suggested that the cause and duration of infertility were two of the most important determinants affecting pregnancy outcomes. Generally, infertile women have more than one reason for using ART. We haven’t compared the incidence of complication between different reasons.

Although the ART is developing increasingly, all of the underlying maternal factors like older age, pelvic inflammatory disease, polycystic ovary syndrome and obesity have a negative impact on pregnancy progress (13, 14). The duration of pregnancy which is called gestational age. Our study showed that the difference in the mean gestational age between the ATR group and NC group was significant and women in ART group delivered earlier than women in NC group (32.35 vs. 36.17 weeks). We still found that the age of women in ART group were significantly older compared with the NC group (32.53 vs. 29.87 years). Older age has always been some of the risk factors for pregnancy complications, such as PE, GDM and so on (15, 16).

Our study evaluates the live birth rate in both of the ART group and NC group. In the ART group, there were 101 cases of patients including missed abortion, complicating with tubal ectopic pregnancy, induced abortion with fetal malformations, and termination of pregnancy with maternal pathological conditions, fetal death and stillbirth. There were 209 cases of patients including the same situations. All of the other cases in each group were live birth. The live birth rate was significantly higher in NC group in our research. Australian In Vitro Fertilization Collaborative Group also reported the high rate of early pregnancy loss, tubal ectopic pregnancies, biochemical pregnancies and spontaneous abortion in a fertilization cohort of 244 pregnancies resulting from in vitro fertilization. 

The high incidence of tubal ectopic pregnancy after in vitro fertilization may be related either to the techniques of embryo transfer or to underlying tubal disease and previous tubal surgery. There are many reasons responsible for high incidence of preterm births and early losses in pregnancy and the reasons are more difficult to determine. Declined function of corpus luteum, underlying diseases, occurrence of complications and multiple pregnancies are the potential factors resulting the preterm births and early losses (17, 18). We didn’t evaluate the preterm birth rate. Preterm birth rate means the radio of live birth cases to total cases. The pregnant women have a strong willingness to rescue the newborn in the assisted reproductive pregnancy. Live birth is defined as there is at least one live-born infant no matter in singleton pregnancy or multiple pregnancies.

Live-born infants involve preterm infants born after 28 weeks and viable infants born before 28 weeks. So the live birth rate can reflect the pregnancy outcome much better. Infertility women need COH therapy before fresh embryo transfer, while the procedure of COH may influence the body’s immune function. The influences include the changes of cellular immunity-humoral immune balance, Th1/Th2 balance and Th17/Treg balance. All of the above can affect the fertilization rate, the cleavage rate and pregnancy success rates and induce the complications related to immunity. So the pregnancy outcomes maybe worsen (19-21).

In this study we found that the difference in the incidence of GDM, PE, and ICP, premature rupture of membranes (PROM) and placenta previa between the two groups was significant. But there was no significant difference in the incidence of birth defect between the two groups. We have already known that GDM, PE, and ICP are sex hormone related diseases and PE or ICP are related to immune system. Our study compared the incidence of GDM, PE, and ICP between the two groups in more detail. First, we compared the incidence of GDM, PE, and ICP between fresh embryo transfer group and frozen-thawed embryo transfer group. Fresh embryo transfer group was divided into three subgroups- long protocol subgroup, short protocol subgroup and ultra-long protocol subgroup according to the different COH program. 

We compared the incidence difference in different subgroups separately. The Other programs subgroup including ultra-shorting protocol, and GnRH-antagonist protocol were not included in the statistical range because of the low number of cases. We compared the incidence difference according to the number of fetuses. Also, we divided the ART group in some subgroups according to the different exogenous progesterone treatment time or dosages of Gn separately and compared the difference in incidence of complication. It is difficult to pinpoint the exact cause for these increased risks. Maternal age, subfertility, and underlying chronic conditions seem to play a major role. It also has been postulated that there may be an inherent difference in the initiation of the chorion formation while the embryo is in vitro, leading to an abnormal placentation in both location and function. We will analyze and discuss the different complications separately.

**GDM**

Gestational diabetes mellitus (GDM), which complicates 3-7% of all pregnancies, is associated with increased maternal and fetal morbidity (22). There was a significant difference in the incidence of GDM between the ART group and NC group (11.21%/6.81%) and the incidence of GDM in ART group was higher in our study. The meta-analyses by Allen and Wilson in 2006 reported up to 2-fold increase in gestational diabetes in pregnancies achieved with ART (23). In the ART group, 12.8% of infertile women complicated with PCOS which provided the basis for insulin resistance. Karen *et al* supported the idea that increased insulin resistance (IR) was associated with the subsequent development of overt glucose intolerance later in pregnancy. So the incidence of GDM in ART group is higher. But this can only explain part of the reason. The use of GnRH-a could cause glucose intolerance during COH and fresh embryo transfer (24). Dickerson *et al* reported that the HOMA-IR levels was higher after COH and they suggested a positive correlation of HOMA-IR levels above a threshold level of 2.5 to total ovarian follicle count following COH in the non-PCOS patients (25). Taşcılar *et al* study also showed an exaggerated elevation in IR in GnRHa-treated ICPP children (24). 

Moreover, exogenous progesterone treatment during early pregnancy has a direct effect on glucose metabolism and causes GDM. Pieard *et al* observed that gestational diabetes coincides with elevated circulating progesterone levels. They also demonstrated an important role of progesterone signaling in insulin release and suggested that it affected the susceptibility to diabetes. Progesterone may play a decisive role on insufficient insulin secretion during pregnancy (26). The onset of GDM is usually in second trimester of pregnancy because of the high levels of progesterone. Couch *et al* found that the level of plasma progesterone in pregnant women with GDM was higher at any time during pregnancy compared to healthy pregnant controls (27). Infertile women use different dosages of hormone during COH and accept ocolytic therapy with exogenous progesterone treatment. All of the above could have an effect on the level of progesterone during the whole pregnancy. So the incidence of GDM after ART will increase.

Furthermore, Chen *et al* reported that serum leptin and follicular fluid leptin increased during COH for in vitro fertilization-embryo transfer (28). Chakrabarti *et al *found elevated leptin response might exert adverse impacts on pregnancy success during in vitro fertilization-embryo transfer possibly by modulating uterine receptivity (29). Leptin regulates the secretion of sex-steroid trough hypothalamic-pituitary-gonadal axis in order to be involved in energy metabolism during pregnancy. Leptin can also regulate the secretion of placental hormones in GDM (30). High plasma leptin level stimulates the secretion of progesterone from trophoblast cells while progesterone increases insulin resistance through decreasing glucose transportor-4 in muscle and adipose tissue. 

High leptin concentration can inhibit the aromatization of androstenedione granulose cells and prevent the transformation of androstenedione into estradiol, which causes the increasing of serum androgen level. The increased activity of androgen affects the secretion of insulin and the internal homeostasis environment of glucose. Leptin is involved in the pathogenesis of GDM through regulating the secretion of estrogen, progesterone, androstenedione and other hormones relating to insulin resistance. All of the above explain the differences in the incidence of GDM between different COH programs and different dosages of Gn. 

The difference in the incidence of GDM with different number of fetus remained significant and multiple pregnancies were a risk factor in GDM. Manisha reported that human placental lactogen is higher in twin pregnancies than singleton pregnancies and that should increase insulin resistance and risk for gestational diabetes mellitus (31). Corrado *et al* found that the decreased insulin sensitivity in pregnancy may be modiﬁed by several factors, such as diet, BMI, maternal age, and the placental mass, all of which may play a role affecting β-cell function and sensitivity to insulin (32). It has been suggested that in multiple pregnancies with two placentas or one that is larger, the incidence of gestational diabetes may be increased (33). 

**PE**

Our data showed that the incidence of PE was higher in ART group compared with NC group (10.51%/5.49%). The meta-analysis in 2004 reported a 1.5 times increased risk of preeclampsia in ART singleton pregnancies, and Schieve *et al* also suggested an increased risk of pregnancy-induced hypertension and preeclampsia in ART conceptions (34, 35). Sazonova *et al* reported significantly increased crude OR for preeclampsia in singleton pregnancies after transfer, including fresh and cryopreservation cycles, when compared with singleton pregnancies in the general population (36, 37). Because of increasing serum leptin and increasing insulin resistance after COH, the incidence of PE could be higher. Amal*et al* reported that maternal serum leptin is significantly elevated in preeclampsia (38). High level of serum leptin could increase insulin resistance. Insulin resistance could increase blood pressure in pregnancy through several mechanisms. Insulin resistance is complicating with lipid metabolism disorders. The level of serum adiponectin can affect the function of vascular endothelial cells. Insulin resistance could reduce the activity of Na^+^/K^+^-ATPase. While the transport and exchange abnormalities of Na^+^-K^+^ could affect the vascular smooth muscle sensitivity to vasoactive substances. D'Ann *et al* found that the HOMA-IR levels were higher in PE group compared to control group (39). 

The using of GnRH-a is necessary in fresh embryo transfer during COH. GnRH distributes among the immune system and has an important role in regulating immune function. GnRH has been proved to be one of the messengers connecting the nervous system, immune system and endocrine system. It was reported that CD3^+^CD25^+^T cells and CD69^+^CD25^+^T cells were activated, while CD4^+^CD25^+^T cells were temporarily suppressed in patients with a successful IVF after using of GnRH-a. So GnRH-a can affect the immune balance. Svoboda *et al* found that the GnRH-a/hCG protocol promoted CD3+/CD8+ and KIR2DL4+ NK cell levels and decreased the embryo transfer success in otherwise fertile women (40). Immune imbalance including Th1/Th2 imbalance and Th17/Treg imbalance is correlated with the occurrence of PE. So we may conclude that there is a strong association between COH and the occurrence of PE.

The incidence of PE was significantly higher in fresh embryo transfer group and frozen-thawed embryo transfer group compared to NC group. There was no significant difference in the incidence of PE between fresh embryo transfer group and frozen-thawed embryo transfer group. Although patients will accept exogenous estrogen to promote the endometrial growth and increase endometrial receptivity after frozen-thawed embryo transfer, it does not affect the incidence of PE. Because the dosage of estrogen is few and the period is short, it does not affect the expression of estrogen receptor in placenta and the level of serum estrogen in the second or third trimester of pregnancy.

We compared the incidence of PE between singleton pregnancies, twin pregnancies and triplet pregnancies. The incidence was highest in triplet pregnancies and lowest in singleton pregnancies. The difference was significant. It is well-established that the risk of preeclampsia is greater in twin rather than in singleton pregnancies and is even greater in triplets (41). Our study is consistent with other studies. An increase in the circulating levels of sFlt1 in the maternal serum is characteristic of preeclampsia and is thought to play a major role in the pathogenesis of this disease. Two hypotheses may explain the connection between multigestational pregnancy and preeclampsia. 

The first hypothesis is that, in multigestational pregnancies, the placenta is programmed to produce more sFlt1 per unit of placenta, perhaps because it is more hypoxic. The second hypothesis is that the genetic message for sFlt1 per unit placenta is not elevated in multigestational placentas but that there is more trophoblastic placental tissue, so the total production of sFlt1 and its concentration in serum are increased (42). In another study, Fox *et al* reported that the risk factors independently associated with preeclampsia were egg donation and pre-pregnancy obesity in twin pregnancy (43).

**ICP**

The result showed that the incidence of ICP was higher in ART group than in NC group. There was no significant difference in the incidence of ICP between fresh embryo transfer group and frozen-thawed embryo transfer group. We also compared the incidence of ICP between singleton pregnancies, twin pregnancies and triplet pregnancies. The incidence was highest in triplet pregnancies and lowest in singleton pregnancies. During the process of COH, the patients accept the injection of GnRH-a, Gn and HCG. They also get exogenous progesterone treatment or exogenous estrogen treatment after embryo transfer. All of the drugs’ metabolisms take place in liver and that will overload the liver. Moreover, other studies reported that there was a correlation between ICP and immune imbalance including Th1/Th2 imbalance and Th17/Treg imbalance (44). Twin and triplet pregnancies, which are associated with higher hormone levels, show a higher incidence of ICP (45).

**Placenta previa**

Our study showed that the incidence of placenta previa was higher in ART group. Verlaenen *et al* study in 1995 reported that placenta previa occurred four times in IVF pregnancies compared with matched controls (46). Romundstad *et al* reported a 6-fold increased risk of placenta previa in singleton pregnancies conceived by ART compared with naturally conceived pregnancies. A substantial proportion of the increased risk may be attributable to ART (47). The placement of embryos in the lower half of the uterine cavity and the myometrial movements arising from the fundus toward the cervix during the early secretory phase could account for the implantation of the embryo in the lower part of the uterus (48).

We did not compare the incidence of placenta previa between fresh embryo transfer group and frozen-thawed embryo transfer group. Some studies have compared the risk of placenta previa in cryopreservation cycles and fresh cycles, and there is some evidence that fresh transfers were associated with an increased risk of placenta previa (49). Sazonova *et al* also found a lower rate of placenta previa in pregnancies from cryopreservation cycles than those from fresh cycles (50).

**PROM**

The incidence of PROM in ART group was higher than NC group. The reasons of infertile show that many infertility patients have a history of uterine surgery which may cause tubal inflammation. The inflammation may be persistent and will be a high risk of PROM. The pressure of the amniotic cavity during twin pregnancies and triplet pregnancies will increase in the second and third trimester of pregnancy. It is also an increased risk of PROM.

**Birth defects**

Birth defects include fetal malformations determined by ultrasound, chromosomal abnormalities and malformation found after birth such as strephenopodia, hexactylia and so on. A meta-analysis of birth defects in children conceived by IVF and ICSI was published in 2012.It reported that there was a significantly increased risk of birth defects in infants conceived by ART, but ICSI did not increase the risk compared with IVF (51). Another study in 2012 shows that treatment with ART is associated with increased risks of cardiovascular, musculoskeletal, urogenital, and gastrointestinal defects and cerebral palsy (52). The effect of ART on the nervous system was relatively obvious compared with the effects on eyes, ears, face, and neck, which may suggest that the earlier developed systems were more sensitive to birth defects by ART. These could be due either to ART procedures themselves or to the underlying infertility in the couples seeking treatment, or to both (53). 

But our study showed that there was no significant difference in the incidence of birth defect between the ART group and NC group. There may be two reasons. One reason is that the sample size is small. Another reason is that many pregnancies with chromosomal abnormalities end with spontaneous abortion in the first trimester. This kind of pregnancy outcome affects the incidence of birth defect. 

**Limitation**

Many patients in the group of more than 45 days’ exogenous progesterone treatment end the pregnancies before 20 weeks because of complete abortion and missed abortion. The pregnancies are hard to continue until late pregnancy. The low live birth rate in this group cannot reflect the rate of GDM, PE or ICP objectively. The cases in this report are all from Hunan province in China and are with regional differences. Some incidences of complications like ICP have obvious regional differences. So part of the data in this report cannot reflect the situation in the world. This study is a clinical observation. Many further experimental studies are still needed to further prove the conclusions.

## Conclusion

Compared with natural conception (NC) group, the risk of adverse maternal outcomes increases in ART populations. The dosages of Gn should be limited. Exogenous progesterone treatment is safe

## Conflict of interest

No potential conflict of interest relevant to this article is reported.
